# Le syndrome du condyle occipital: la partie visible de l'iceberg

**DOI:** 10.11604/pamj.2019.33.268.19280

**Published:** 2019-07-29

**Authors:** Amine Raggabi, Issam Lalya, Ahmed Bourazza

**Affiliations:** 1 Hôpital Militaire d’Instruction Mohammed V, Rabat, Maroc; 2 Faculté de Médecine et de Pharmacie, Université Cadi Ayyad, Marrakech, Maroc

**Keywords:** Occipital condyle syndrome, imaging, investigation for primary tumor, symptomatic treatment, etiologic treatment, Occipital condyle syndrome, imaging, investigation for primary tumor, symptomatic treatment, etiologic treatment

## Abstract

Le syndrome du condyle occipital est une entité clinique rare, définie cliniquement par l'association de céphalées occipitales intenses et d'une paralysie de la douzième paire crânienne. Son étiologie est dominée par la pathologie tumorale métastatique. L'imagerie est la pierre angulaire du diagnostic permettant à la fois de mettre en évidence la lésion du condyle occipital et de rechercher la tumeur primitive. Le traitement repose d'une part sur le soulagement de la symptomatologie douloureuse moyennant les antalgiques, les corticoïdes et très souvent la radiothérapie externe. Et d'autre part sur le traitement de la tumeur métastatique par chimiothérapie cytotoxique, thérapies ciblées ou immunothérapie en fonction du profil moléculaire de la tumeur primitive.

## Abstract (en)

Occipital condyle syndrome is a rare clinical disorder, clinically defined by the association of intense occipital headaches and paralysis of the twelfth paired cranial nerve. Its etiology is dominated by metastatic tumor. Imaging is the gold standard for diagnosis allowing to highlight occipital condyle lesion as weel as to find out primary tumor. Treatment is based on pain relief using analgesics, corticosteroids and very often, external radiotherapy. On the other hand, treatment of metastatic tumor is based on cytotoxic chemotherapy, targeted therapies or immunotherapy depending on the molecular profile of the primary tumor.

## Introduction

Le syndrome du condyle occipital est une entité rare associant des céphalées occipitales intense et une paralysie du XII. Les étiologies sont dominées par la pathologie tumorale surtout métastatique mais pas exclusivement. Nous rapportons l'observation d'un cas où ce syndrome a permis de révéler une métastase intracrânienne secondaire à un cancer pulmonaire.

## Patient et observation

**Figure 1:**
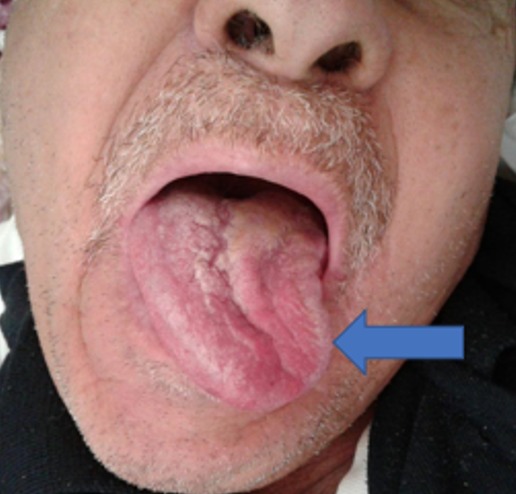
Déviation de la langue témoignant d’une paralysie du XII

**Figure 2:**
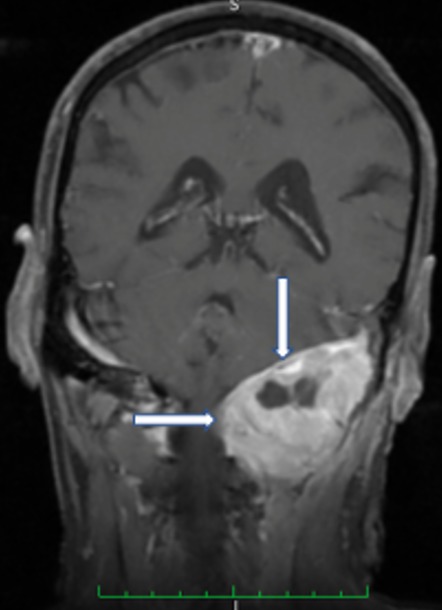
IRM en coupe coronale montrant une lésion du condyle occipital gauche

**Figure 3:**
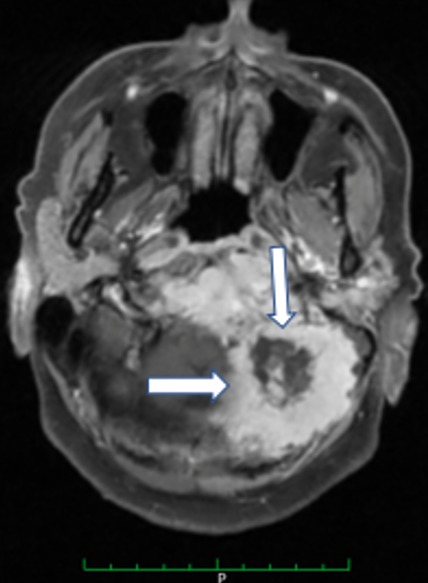
IRM en coupe axiale montrant une lésion du condyle occipital gauche

Patient de 67 ans, sans antécédents notables excepté un tabagisme chronique estimé à 40 PA, a été admis pour des céphalées occipitales récentes (moins de 10 jours) et intenses, associées à une dysarthrie. Ces céphalées intenses à recrudescence nocturne irradiaient vers l'oreille gauche et étaient résistantes aux antalgiques habituels. L'examen clinique objectivait une paralysie franche du XII gauche (Figure 1), sans autres signes neurologiques (pas d'autres déficits sensitivomoteurs, pas de syndrome méningé). Les artères temporales étaient normalement battantes et le reste de l'examen notamment ORL et général étaient normaux. Il n'y avait pas de syndrome inflammatoire biologique en faveur d'une maladie d'Horton, les autres examens biologiques (NFS, Ionogramme, bilan hépatique, EPP) étaient normaux. Une écho doppler des troncs supra aortiques était normale éliminant une dissection carotidienne. Une IRM cérébrale était réalisée d'emblée et montrait une lésion de grande taille du tissu spongieux du condyle occipital gauche avec extension à l'angle ponto-cérébelleux, évoquant plutôt une métastase avec hypo signal T1, hyper signal T2, rehaussé de façon homogène et intense à l'injection de gadolinium (Figure 2, Figure 3). Un bilan à la recherche d'une néoplasie profonde fut réalisée, permettant de découvrir à la TDM thoraco-abdomino-pelvienne une tumeur de l'apex pulmonaire gauche avec des métastases au niveau des deux champs pulmonaires et au niveau hépatique. Une biopsie scanno-guidée fut réalisée dont l'étude anatomopathologique et immunohistochimique était en faveur d'adénocarcinome pulmonaire (EGFR muté, ROS1 et ALK négatifs). Le patient a bénéficié d'un traitement médical associant antalgiques palier 2 et corticoïdes, et d'une radiothérapie encéphalique totale à la dose de 30 Gy en 10 fractions de 3 Gy, 5 fractions par semaine, moyennant les photons 6MV d'un accélérateur linéaire. L'évolution sous traitement était spectaculaire avec quasi disparition des douleurs. Le patient est actuellement sous traitement par inhibiteur tyrosine kinase anti-EGFR par voie orale, avec une bonne évolution.

## Discussion

Le syndrome du condyle occipital (SCO) est une entité rare, il associe des douleurs occipitales intenses et une paralysie du XII, et survient surtout dans un contexte métastatique [1-5]. La première description a été faite par Greenberg en 1981 [1], et puis deux séries ont principalement étudié cette entité, celle de Capobianco en 2002 portant sur 11 patients, et celle de Moris en 1998 portant sur 4 patients. Les caractéristiques sémiologiques sont l'association constante de douleurs occipitales intenses et l'atteinte du XII. La douleur occipitale est intense, continue, surtout unilatérale, avec une sensibilité à la palpation de la région endolorie et parfois une irradiation mastoïdienne, frontale ou au vertex. Une majoration par les mouvements de flexion de la nuque ou de rotation de la tête est assez fréquente. Parfois, une attitude particulière de la tête en rotation du coté douloureux et immobilisation avec les mains est assez fréquente [3], pouvant mimer un simple torticolis. La deuxième caractéristique principale constitutive de ce syndrome est l'atteinte du XII, souvent précédée par la symptomatologie douloureuse de plusieurs jours à quelques semaines, pouvant alors être confondue avec des céphalées de tension ou une névralgie d'Arnold, retardant ainsi l'exploration par l'imagerie. Cette atteinte du XII entraine toujours une légère dysarthrie qui peut être objective ou fonctionnelle, parfois associée à une dysphagie. L'imagerie constitue le temps fort du diagnostic de ce syndrome, permettant de confirmer la lésion du condyle occipital avec un meilleur apport de l'IRM par rapport au scanner. Elle doit comporter les séquences T1, T2, FLAIR, l'injection de gadolinium, mais aussi les séquences FAT-SAT avec saturation du signal de la graisse, pour une meilleure étude des tissus spongieux [6]. Classiquement la lésion est en hypo signal T1, hyper signal T2 et FLAIR, avec rehaussement à l'injection de produit de contraste. Cet aspect IRM a été retrouvé chez notre patient avec une prise de contraste intense et homogène. Les étiologies du SCO sont largement dominées par la pathologie tumorale surtout métastatique de cancer cérébral, ORL mais aussi pulmonaire ou digestive. Il peut s'agir soit de tumeurs primitives déjà connues, soit ce syndrome est la manifestation initiale permettant de révéler une tumeur métastatique. Chez notre patient le SCO était la manifestation initiale et a permis de révéler un adénocarcinome pulmonaire métastatique. D'autres cas cliniques ont mis en évidence d'autres étiologies plus rares comme une tuberculose de la jonction cranio-vertébrale [7, 8], avec un meilleur pronostic puisque accessible au traitement anti bacillaire. Enfin, la prise en charge et le pronostic dépendent grandement de la lésion sous-jacente.

## Conclusion

Le SCO est une entité rare, sa connaissance doit inciter à le rechercher car il peut être confondu avec un syndrome douloureux de la face ou une céphalée de tension, et au vu de la grande fréquence des métastases révélées par ce syndrome, parfois inaugurales, la recherche de néoplasie profonde surtout extra-neurologique doit être rapidement et soigneusement faite.

## Conflits d’intérêts

Les auteurs ne déclarent aucun conflit d'intérêts.
